# Dysfunctions in Infants’ Statistical Learning are Related to Parental Autistic Traits

**DOI:** 10.1007/s10803-021-04894-0

**Published:** 2021-02-13

**Authors:** Bettoni Roberta, Valentina Riva, Chiara Cantiani, Elena Maria Riboldi, Massimo Molteni, Viola Macchi Cassia, Hermann Bulf

**Affiliations:** 1 Department of Psychology, Università degli Studi di Milano-Bicocca, Piazza Ateneo Nuovo, 1 (U6), 20126 Milano, Italy; 2 NeuroMi, Milan Center for Neuroscience, Milano, Italy; 3 Child Psychopathology Unit, Scientific Institute, IRCCS E. Medea, Bosisio Parini, Lecco, Italy

**Keywords:** Visual statistical learning, Autistic traits, Infants, Early marker

## Abstract

Statistical learning refers to the ability to extract the statistical relations embedded in a sequence, and it plays a crucial role in the development of communicative and social skills that are impacted in the Autism Spectrum Disorder (ASD). Here, we investigated the relationship between infants’ SL ability and autistic traits in their parents. Using a visual habituation task, we tested infant offspring of adults (non-diagnosed) who show high (HAT infants) versus low (LAT infants) autistic traits. Results demonstrated that LAT infants learned the statistical structure embedded in a visual sequence, while HAT infants failed. Moreover, infants’ SL ability was related to autistic traits in their parents, further suggesting that early dysfunctions in SL might contribute to variabilities in ASD symptoms.

Statistical Learning (SL) is a domain-general mechanism that allows us to learn the statistical information embedded in a continuous stream of elements without awareness or intention (Saffran et al. 1996). Starting from birth (Bulf et al. 2011; Teinonen et al. 2009), infants use SL to extract the statistical structure of a variety of stimuli, such as speech sounds (e.g. Saffran et al. 1996), non-speech sounds (e.g. Saffran et al. 1999), visual shapes (e.g. Kirkham et al. 2002), and actions (e.g. Monroy et al. 2017). This early sensitivity to the input's statistical property is relevant for language acquisition and social understanding. For example, after brief exposure to a string of speech sounds in which there are no definitive acoustic cues for word boundaries, 8-month-old infants can segment different words relying on transitional probabilities between syllables (higher within terms than between words; Saffran et al. 1996; Aslin et al. 1998). Moreover, infants can learn that some action groupings are more likely to occur than others by segmenting the motion flow into units based on the sequence observed actions' sequential predictability (Baldwin et al. 2008).

These findings provide evidence that infants can use statistical computations to develop language skills (Saffran et al. 1996) and build expectations about others' actions and intentions (Hunnius and Bekkering 2014; Ruffman et al. 2012). Starting from this evidence, it has been recently proposed that impairments in SL abilities might be involved in the behavioral endophenotype of Autism Spectrum Disorder (ASD; e.g Saffran 2018; Sinha et al. 2014), a neurodevelopmental disorder that has social-communicative deficits as its major behavioral manifestation, in addition to restricted interests and stereotyped behaviors (APA 2013). To date, studies investigating SL abilities in individuals with ASD have obtained mixed results. Some reported intact SL abilities in ASD children (e.g. Mayo and Eigsti 2012; Roser et al. 2015; see Foti et al. 2015 and Obeid et al. 2016 for reviews), while others reported atypical neural correlates of such abilities (Jeste et al. 2015; Scott-Van Zeeland et al. 2010).

Mayo and Eigsti (2012) tested the ability to track transitional probabilities embedded in speech sequences in high-functioning children with ASD and a control group of typically developing (TD) children, showing that the two groups did not differ in their performance. In line with this evidence, it has also been reported that ASD children's performance did not differ from that of TD controls in tasks where they were requested to extract the statistical structure embedded in multi-shapes visual arrays composed of pairs of shapes with high or low covariation (Roser et al. 2015). Even though children with ASD and TD showed similar behavioral performance on the SL tasks, evidence from electrophysiological and neuroimaging studies have shown differences in the neural processing of the statistical information embedded in the stream between TD and ASD individuals. For example, in an ERP study, Jeste et al. (2015) found that ASD children showed reduced evidence of SL and novelty discrimination compared to TD controls and that heterogeneities in electrophysiological markers of visual SL are associated with individual differences in non-verbal cognition and adaptive social skills. Accordingly, an fMRI study by Scott-Van Zeeland and colleagues (2010) revealed a different activation pattern in high-functioning ASD children compared to TD children during an SL task with speech sequences. These pieces of evidence reveal that SL mechanisms may not be entirely intact in the ASD population when measured at a neural level, and it is in line with the idea that children with neurodevelopmental disorders might show good behavioral performances on a task, but different neural mechanisms relative to TD children (Karmiloff-Smith 2009).

The presence of an SL impairment in ASD is further confirmed by a recent ERP study that has tested infants at familial risk to develop ASD by virtue of having a sibling with a diagnosis (Marin et al. 2020). Using a visual SL task in which infants were presented with sequences of shapes, the authors reported an atypical neural activation in infant siblings of ASD children compared with TD infants. This finding demonstrates the presence of an association between an atypical sensitivity to the statistical property of the input and familial risk for ASD, suggesting that dysfunction in the processing of SL information can be found not only in individuals with a clinical diagnosis of ASD but also in their genetic relatives, in line with evidence that ASD is highly heritable, with heritability estimates ranging between 64 and 91% (Tick et al. 2016).

Recent studies have shown that genetic susceptibility is not confined to the ASD condition but can also be found for a broader range of social and communicative subclinical features, defined as autistic traits, similar to those seen, in more severe forms, in individuals with a diagnosis of ASD (e.g. Bralten et al. 2017; Costantino et al. 2006; Dawson et al. 2002; Gaugler et al. 2014). Autistic traits are continuously distributed in the general population, representing the so-called Broader Autism Phenotype (BAP), and are heritable, with heritability rates ranging from 36.0% to 87.0% (Robinson et al. 2011; Ronald and Hoekstra 2011). It has been recently shown that autistic traits in parents are associated with both the clinical and subclinical behavioral autistic phenotype of offspring (De la Marche et al. 2015; Gerdts and Bernier 2011; Jones et al. 2017; Levin-Decanini et al. 2013; Maxwell et al. 2013; Riva et al. 2019; Schwichtenberg et al. 2010). For example, the incidence of high autistic traits in ASD parents ranges from 2.6% to 80.0% (see review by Rubenstein and Chawla 2018), and autistic traits in ASD parents are associated with the severity of ASD symptoms in children (e.g. Gerdts and Bernier 2011; Levin-Decanini et al. 2013; Maxwell et al. 2013). Moreover, children of parents with high autistic traits are more likely to develop BAP phenotypical characteristics (Riva et al. 2019; Rubenstein et al. 2018). This suggests that autistic traits might be useful to further understand and describe both clinical and subclinical conditions (for more details see Ruzich et al. 2016).

An emerging area of research showed that some dysfunctions underlying the ASD phenotype are also evident in individuals who possess high levels of autistic traits in the general population (Baron-Cohen et al. 2001; Grinter et al. 2009). For example, high autistic traits are associated with abnormalities in neural activity and behavioral difficulties in areas that overlap with those that are impaired in ASD, such as emotion recognition (McKenzie et al. 2018), face processing (Stavropoulos et al. 2018), multisensory integration (van Laarhoven et al. 2019) and learning of implicit social rules (Hudson et al. 2012). Moreover, higher levels of autistic traits in preschool-age children predict later delays in cognitive and language functioning (Möricke et al. 2010). Finally, infants whose fathers have high autistic traits show atypical visual attention abilities (Jones et al. 2017; Ronconi et al. 2014) and alpha-band brain oscillations (Riva et al. 2019), suggesting the presence of an association between parental autistic traits and the functioning of cognitive processes in their offspring.

In light of earlier demonstrations that ASD-related traits vary in the general population and correlate with a genetic predisposition towards ASD (Wheelwright et al. 2010), it is plausible to hypothesize the presence of an association between infants' SL abilities and the intensity of sub-clinical traits in their parents. Dysfunctions in SL mechanisms have been linked to ASD symptoms (Jeste et al. 2015; Marin et al. 2020) and ASD traits (Parks et al. 2020; Stevenson et al. 2017), pointing to the idea that perturbations in SL mechanisms might depend on the variation of ASD sub-symptoms severity. The investigation of SL in infants with parental BAP appears important to evaluate the potential relationship between SL and the development of subclinical social impairments. Indeed, emerging research suggests that SL plays a crucial role in social and communicative skills as it allows to find regularities in social interactions and, in turn, to adapt to the social environment (e.g. Baldwin et al. 2008; Parks et al. 2020).

Capitalizing on this evidence, the present study aimed to investigate the functioning of visual SL mechanisms in 7-month-old infants offspring of non-diagnosed adults who show high vs. low subclinical autistic traits. Visual SL abilities were assessed using the visual habituation task developed by Kirkham et al. (2002) in two groups of infants, i.e., infants whose parents showed high autistic traits (HAT group) and infants whose parents showed low autistic traits (LAT group). Infants were habituated to a sequence of visual shapes organized in a statistical structure and were subsequently presented with six test trials alternating the familiar sequence and a new sequence in which shapes were presented in random order. If infants were able to extract the statistical information embedded in the habituation sequences, they would look longer at the new test trials than at the familiar ones. Parental autistic traits were measured through the Autism Quotient (AQ) self-report questionnaire developed by Baron-Cohen et al. (2001). The AQ questionnaire is widely used to quantify the severity of autistic traits in both general and clinical populations (Ruzich et al. 2016). It has high test–retest reliability and internal consistency (Baron-Cohen et al. 2001), as well as cross-cultural validity (Ruta et al. 2012). We expected to observe significant differences in looking time behavior over the test trials between LAT and HAT infants, with only LAT infants showing a significant novelty preference. Within the dimensional view of autistic traits, we move beyond the investigation of group differences to explore the link between individual differences in infants' SL abilities and parental autistic traits.

## Method

### Participants

The sample included a total of 46 healthy full-term 7-month-old infants (mean = 6.93 months *SD* = 0.53; 26 males), who were assigned to the LAT group (N = 23) or the HAT group (N = 23) based on their parents' scoring on the Autism Quotient (AQ) questionnaire (Baron-Cohen et al. 2001). The numerosity of our sample was based on a priori power analysis (Faul et al. 2007) that estimated a sample size of 46 participants to obtain a medium effect size of 0.25 with a α = 0.05 and power = 0.90. Data from 7 additional infants were excluded from the final sample because of fussiness. Parents filled in a questionnaire aimed to collect socio-demographic information, and infants' cognitive skills were assessed using the Bayley Scales of Infant Development (Bayley 2006). Inclusion criteria were gestational age above or equal to 36 weeks, birth weight above or equal to 2500 gr, and a Bayley cognitive score above or equal to 7. The two groups of infants did not differ in demographics (e.g. socioeconomic status and parental age), individual and cognitive characteristics (e.g. gestational weeks, birth weight, and Bayley cognitive subscale), as reported in Table 1. The study was approved by the Ethical and Scientific Committees of the Eugenio Medea Scientific Institute and the University of Milano-Bicocca, and has been conducted in accordance with the ethical standards of the 1964 Helsinki Declaration. Parents gave their informed written consent for their infant's participation.

**Table 1:** Characteristics of the LAT and HAT groups of infants

Variables	LAT group		HAT group		t-test		
	Mean	SD	Mean	SD	t (df)	*p*	Cohen's d
Gestational age (weeks)	38.82	1.40	38.91	1.11	− 0.24 (42)	0.813	− 0.07
Birth weight (grams)	3357.73	475.28	3297.27	413.99	0.45 (42)	0.655	0.14
Bayley Cognitive sub-scales^a^	11.86	1.11	11.55	1.47	0.78 (41)	0.439	0.24
Mother's age (years)	33.78	4.12	33.70	5.51	0.06 (44)	0.952	0.02
Father's age (years)	35.91	4.67	34.43	9.29	0.68 (44)	0.499	0.21
SES^b^	58.48	12.19	57.75	18.53	0.15 (41)	0.878	0.05
Parental AQ (raw-scores)	16.48	2.88	25.00	3.78	− 8.70 (44)	< .001	2.56

Independent samples t-tests were run to compare infants’ demographic, individual and cognitive characteristics between the LAT and HAT group of infants. (a) Infants’ cognitive abilities were assessed using the Cognitive subscale from the Bayley Scales of Infant Development (Bayley 2006); (b) Socio-economic status was assessed using the Hollingshead scale, which scores ranges from 10 to 90. The score was assigned to each parental job, and the higher score was used when both parents were employed (Hollingshead 1975)

### Procedure

#### Assessment of Parental Autistic Traits

One month prior to the infant's 6^th^ month birthday, each parent completed the Italian version of the AQ questionnaire (Ruta et al. 2012). The questionnaire includes 50 items grouped in 5 subscales: social skills, attention switching, attention to detail, communication, and imagination. The total AQ score ranges from a minimum of 0 to a maximum of 50 and is calculated by adding up all of the scores from the different subscales, with higher scores indicating higher autistic traits. Infants were divided into two groups based on the total AQ score obtained by each of their parents using the broader autism phenotype cut-off of 1 SD from the standardized mean, which corresponds to 21 for males and 20 for females, Ruta et al. (2012). Infants whose parents both scored below the cut-off were included in the LAT group, while infants who had at least one parent who scored equal or above the cut-off were included in the HAT group.

The total AQ scores were calculated on the higher score obtained by one of the two parents. Among the 23 HAT infants, 13 (56.53%) were included in the group based on the father AQ score, 9 (39.13%) were included based on the mother's AQ score, and 1 (4.34%) based on the AQ score of both parents. These data show that high autistic traits in our sample were more consistent in fathers than mothers, which is in line with previous evidence that BAP traits tend to aggregate more often in male relatives than female relatives (Wheelwright et al. 2010; Pickles et al. 2000). The mean AQ score for the LAT group was 16.48 (*SD* = 2.88), which differed from the mean AQ score for the HAT group (*M* = 25, *SD* = 3.78), *t*(44) = -8,70, *p* < 0.001 (Table 1). These values resembled the normative data of the BAP in the general population (*M* = 15.77, *SD* = 6.75) and in the ASD population (*M* = 25.97; *SD* = 8.09; Fusar-Poli et al. 2020). The mean maternal AQ score was 13.35 (*SD* = 3.18; range = 6–19) in the LAT group and 17.50 (*SD* = 6.69; range = 7–30) in the HAT group, the mean paternal AQ score was 15.13 (*SD* = 3.74; range = 8–20) in the LAT and 22.95 (*SD* = 5.40; range = 14–36) in the HAT group.

#### Assessment of Infants' SL and Cognitive Abilities

**Fig.1:**
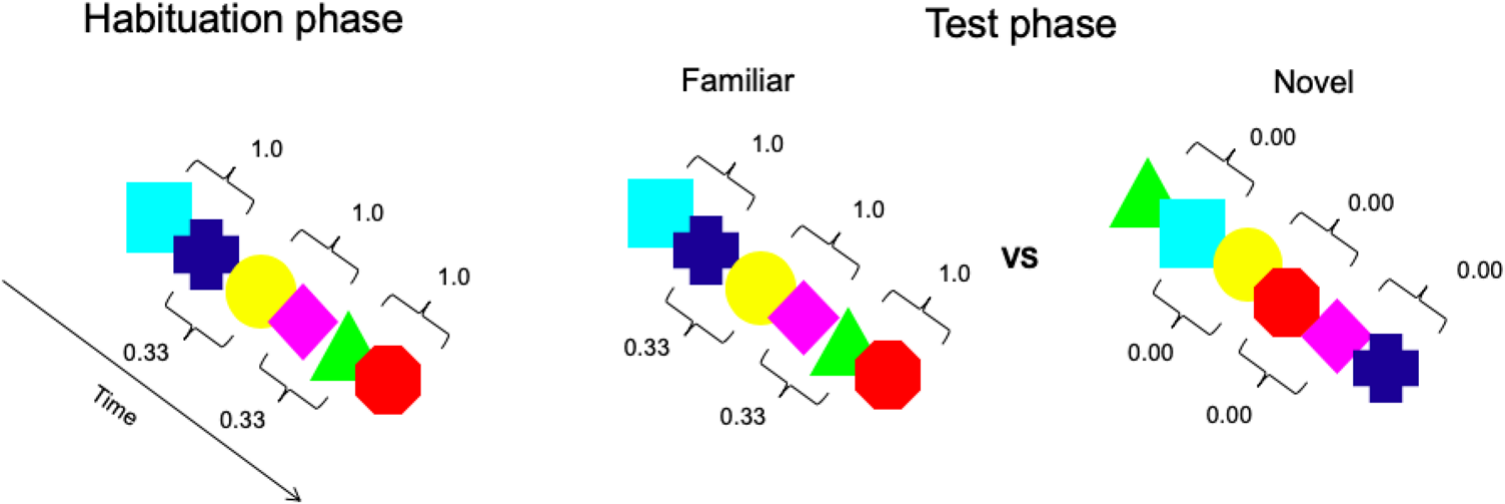
Schematic representation of the visual SL task

An infant-controlled habituation task was used to assess infants' visual SL abilities. Following Kirkham et al. (2002), stimuli were six colored shapes (turquoise square, blue cross, yellow circle, pink diamond, green triangle, and red octagon) presented one at a time in a continuous stream in the center of the screen, without breaks or delay between them (Fig. 1). Each shape remained on the screen for 750 ms and loomed along both the vertical and horizontal axes from 3 to 10 cm. Stimuli were generated using E-prime 2.0 software and presented on a 21-inch monitor with a resolution of 1280 × 720 pixels on a black background. The six shapes were used to generate a habituation sequence and a test sequence. In the habituation sequence, the shapes were organized into three pairs that were presented in random order. This way, the transitional probability between shapes was 1.0 within each pair and 0.33 between pairs (Fig. 1). In the test sequence, the 6 shapes were presented in random order, with the only constraint that no more than two identical shapes could appear in a row. As a result, the transitional probability between the shapes in the novel sequences was 0. Looking time (s) towards the test sequences was used as the dependent variable.

Each trial started with the appearance in the center of the screen of a cartoon animated image associated with varying sounds, which served as an attention-getter. As soon as the infant fixated on the screen, the experimenter turned off the cartoon and started the sequence presentation. Each trial continued until the infant looked at the sequence s for a minimum of 500 ms and ended when the infant looked away for 2 s or looked for a maximum of 60 s. The habituation sequence was presented until the infant viewed 12 trials or met the habituation criterion (a 50% decline in looking time on three consecutive trials, relative to the looking time on the first three trials). Following the habituation phase, infants viewed six test trials in which the habituation and the test sequences were presented in alternation, with the order of presentation counterbalanced across participants.

Infants were tested in a sound isolated and dark silent-cabin while seated on their parent's laps at approximately 60 cm from the stimulus presentation monitor. The parent was instructed not to interact with the infant, and he/she was naïve to the research hypothesis. A video camera positioned just above the monitor recorded the infant's face and sent a visual input to another computer monitor, allowing the online coding of infants' looking times by an experimenter who was blind to the stimuli presented. The image of the infant's face was also recorded via a Mini-DV digital recorder for the purpose of offline coding. For about half of the participants (N = 20), looking times during test trials were coded offline by a second independent observer who was blind to the experimental condition the infants were tested in. Inter-observer agreement between the two observers who coded the data live or from a digital recording, as computed on total fixation times on each of the six test trials, was *r* = 0.95 (*p* < 0.001, Pearson correlation). Online coded looking times were used as the dependent variable in the analyses.

At the end of the habituation task, the experimenter administered the Cognitive subscale of the Bayley Scales of Infant Development (Bayley 2006).

## Results

### SL Task

#### Habituation Phase

**Fig. 2:**
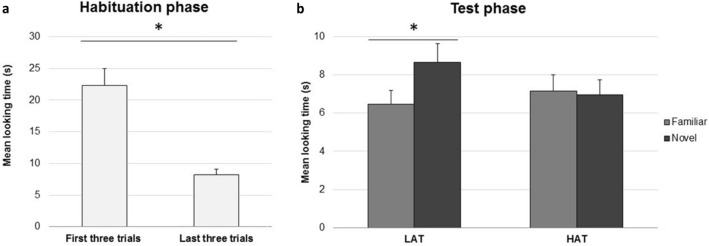
**a** Mean total looking time (+ SE) to the first three and last three habituation trials. **b** Mean total looking time (+ SE) to familiar and novel test trials in the LAT and HAT group of infants. * p < .05

All infants reached the habituation criteria. Two independent-sample t-tests revealed that the LAT and the HAT groups did not differ in their total habituation times (LAT: *M* = 98.73 s, *SE* = 10.65; HAT: *M* = 120.79 s, *SE* = 17.59), *t*(44) = − 1.073,* p* = 0.289, *d* = − 0.316, nor in the number of trials to habituate (LAT: *M* = 6.74, *SE* = 0.25; HAT: *M* = 8.04, *SE* = 0.76), *t*(44) = − 1.637, *p* = 0.114*, d* = − 0.481. A 2 (group: HAT, LAT) × 2 (habituation trial: first three, last three) Analysis of Variance (ANOVA) revealed an overall significant decline in mean looking times between the first three (*M* = 22.35 s, *SE* = 2.65) and the last three habituation trials (*M* = 8.25 s, *SE* = 0.82), *F*(1,44) = 47.521, *p* < 0.001, *ƞ*^*2*^_*p*_ = 0.519. No other main effects or interactions were significant (*p*_*s*_ > 0.69) (Fig. 2).

#### Test Phase

To determine whether infants were able to discriminate between the familiar and novel test sequences, we performed an ANOVA on looking times during the test trials with group (HAT, LAT) and test trial order (familiar-novel, novel-familiar) as between-subjects factors, and test trial pair (first, second, third) and test trial type (novel, familiar) as within-subjects factors. The analysis revealed a main effect of test trial pair, *F*(2,84) = 5.266, *p* = 0.007, *ƞ*^*2*^_*p*_ = 0.11, as infants’ looking times were overall longer in the first trial pair (*M* = 8.64 s; *SE* = 0.76) than in the second pair (*M* = 6.61 s; *SE* = 0.52; Bonferroni corrected *p* = 0.014). Moreover, the analysis revealed a significant Group x Test Trial Type interaction, *F*(1,42) = 4.567, *p* = 0.038, *ƞ*^*2*^_*p*_ = 0.098, and a Group x Test Trial Type x Test Trial Pair interaction, *F*(2,84) = 5.298, *p* = 0.007, *ƞ*^*2*^_*p*_ = 0.112. No other effects attained significance (*ps* > 0.06).

To further explore these interactions, we performed two additional ANOVAs, one for each group of infants, including test trial pair (first, second, third) and test trial type (novel, familiar) as within-subjects factors. For the LAT group, the ANOVA confirmed the presence of a significant Test Trial Pair main effect, *F*(2,44) = 6.041, *p* = 0.005, *ƞ*^*2*^_*p*_ = 0.215, which was due to a decline in infants’ looking times from the first (*M* = 9.45 s, *SE* = 1.20) to the third trial pair (*M* = 5.96 s, *SE* = 0.88; Bonferroni corrected* p* = 0.026), indicating a weariness effect in the last test trial. The ANOVA also revealed a main effect of test trial type, *F*(1,22) = 7.456, *p* = 0.012, *ƞ*^*2*^_*p*_ = 0.253, as infants looked overall longer to the novel test sequences (*M* = 8.24 s, *SE* = 0.99) than to the familiar ones (*M* = 5.97 s, *SE* = 0.60). No other effects attained significance (*ps* > 0.08). The ANOVA performed on the HAT group did not reveal any significant effect or interaction (all *p*s > 0.09) (Fig. 2). In particular, not the Test Trial Type main effect, *F*(1, 22) = 0.273, *p* = 0.607, nor any interaction involving this factor (*p*s > 0.09), attained significance.

#### Relationship Between Infant's SL Abilities and Parent's Autistic Traits

**Fig.3:**
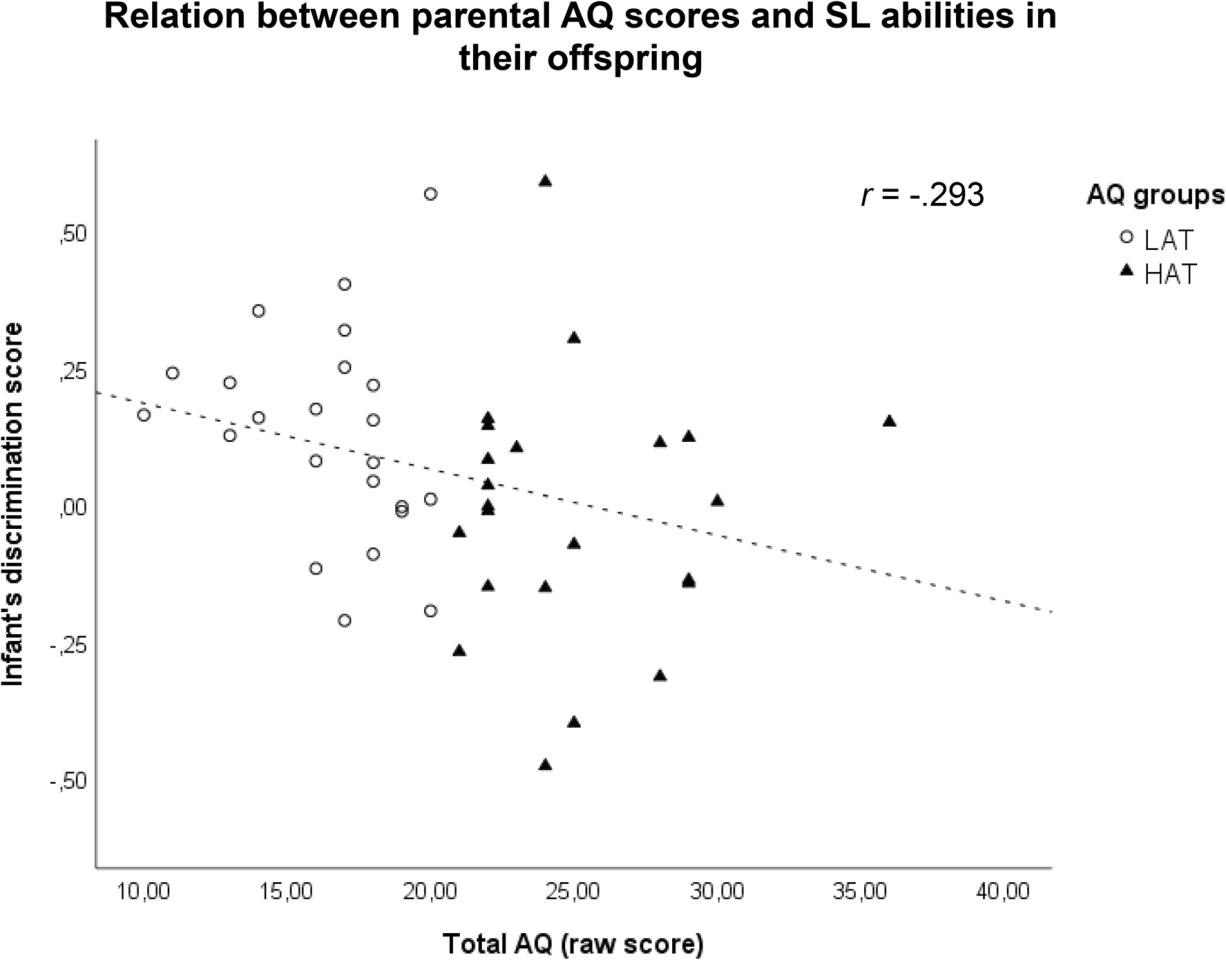
Correlation plot of the relationship between the parental AQ scores and infant’s discrimination score in SL task for both groups LAT and HAT

To provide a further test of the association between parental autistic traits and infants' SL abilities, we conducted a series of Pearson correlational analyses including parental total AQ scores and subscale scores and infants' performance at discriminating between the novel and familiar test trials (discrimination score) during the SL task. We selected the higher among the parental AQ total and subscale scores for each infant and obtained a discrimination score by computing the proportional difference between the total looking time to the novel test trials and total looking to on the familiar test trials (N - F/N + F). The analyses revealed a negative association between discrimination scores and parent total AQ scores, *r*(46) = − 0.293, *p* = 0.048 (Fig. 3), and a negative association between discrimination scores and the AQ social skills subscores, *r*(46) = − 0.303, *p* = 0.041. After Bonferroni correction (α/5 = 0.01) the relationship between discrimination scores and the AQ social subscale did not reach the significance.

## Discussion

The present study aimed to investigate the functioning of visual SL in infants at risk to develop ASD clinical or subclinical features by virtue of having a parent with high autistic traits. Using a visual habituation task, 7-month-old infants whose parents had high (HAT group) and low (LAT group) autistic traits were tested for their ability to discriminate between a sequence of abstract visual shapes that contained statistical regularities vs. a non-structured sequence. The use of abstract visual shapes, rather than social or linguistic stimuli, allowed us to examine the functioning of the SL mechanism per se, independently of potential confounds related to the heterogeneity in the participants' (dis)abilities in the linguistic and social domains. Results showed that infants in the LAT group were able to learn the statistical information embedded in the habituation sequences and to discriminate the statistically structured habituation sequence from a random one. In contrast, infants in the HAT showed no discrimination abilities. It should be noted that the criterion to be part of the HAT group was to have at least one parent with a high AQ total score. The literature on BAP in undiagnosed family members of individuals with ASD indicates that high sub-clinical autism-related traits are more frequently observed in one parent than in both parents (Wheelwright et al. 2010). Accordingly, in the current study, only one infant in the HAT group had both parents with high AQ scores (1 SD above the mean). Unfortunately, this prevented us from testing whether having both parents with high autistic traits have an additive effect on infants' ability to extract statistical regularities from sensory input. This question remains to be tested in future studies involving a larger sample size.

The lack of evidence for SL in the HAT group is particularly relevant considering that the statistical structure of the visual sequence was provided by multiple cues, i.e. co-occurrence frequency and transitional probability. Co-occurrence frequency is defined as the rate at which two items (X and Y) appear consecutively in a sequence, whereas transitional probability is the conditional probability of item Y following item X given that X has already appeared, which provides a measure of the strength with which X predicts Y. Previous studies investigating the independent contribution of co-occurrence frequency and transitional probability to SL have shown that, when these cues converge in defining the statistical structure of the input, infants are facilitated in segmenting continuous streams of visual items (e.g. Aslin et al. 1998; Slone and Johnson 2015). Even in the presence of this redundant information, 7-month-old HAT infants in the current study did not show successful discrimination at the test between structured and non-structured shape streams at test.

Several studies have documented an atypical (slower) habituation process in response to repeated auditory and visual stimuli in ASD (i.e. Ruiz-Martínez et al. 2020; Vivanti et al. 2018; Webb et al. 2010), suggesting that ASD children might struggle in creating coherent mental representations of environmental stimuli. However, in the present study, we found that HAT and LAT infants did not differ in their habituation looking times and the number of trials to habituate. Despite that, habituation in HAT infants did not entail the extraction of the statistical structure of the input to the point of allowing them to recognize the familiar structure at the test. One possibility is that HAT infants were not able to build a stable representation of the learned stimulus because of their poor memory capacities. Indeed, deficits in visual working memory are often reported in ASD individuals (e.g. Wang et al. 2017) and in the presence of high autistic traits in undiagnosed individuals (Richmond et al. 2013), and memory constraints modulate SL performance in both adults (e.g. Thiessen 2011) and infants (e.g. Bulf et al. 2011; Vlach and Johnson 2013). Another possible interpretation of HAT infants' failure at discriminating between the familiar and the novel test stimuli relies on their possible difficulty in generalizing the statistical structure extracted from the habituation sequences to a different context. Children with ASD have an exaggerated perception of changes in the environment, and a recent study showed that they are slow to habituate to a repeating stimulus when presented within a changing context (Vivanti et al. 2018). In the current study, infants were repeatedly exposed to a continuous and unchanging stream of shapes engendering the same statistical structure during habituation, while at the test, they viewed the familiar structure intermixed with a novel one, as familiar and novel trials were presented in alternation. This may have made it difficult for HAT infants to recognize the familiarity of the habituation structure, which may have appeared to them as novel as the one embedded in the truly novel sequence. Future studies are needed to examine further the role of habituation and memory in response to novelty in infants at risk for ASD.

Overall, our data provide the first behavioral evidence of atypical SL in infants at risk for BAP by virtue of having a parent who shows high autistic traits. This finding is in line with those showing SL impairment in ASD children (Jeste et al. 2015; Scott-Van Zeeland et al. 2010) and infant siblings of children with ASD (Marin et al. 2020) and confirms the importance of using implicit measures to understand the functioning of SL processes in typical and atypical population. Indeed, in studies where the ability to discriminate between statistically structured and non-structured streams of stimuli was tested in a task requiring participants' explicit responses, ASD adults and children performed just like TD individuals (e.g. Foti et al. 2015), possibly because the recruitment of top-down strategies allowed them to compensate the SL impairments (see Ullman and Pullman 2015).

Since the ability to track statistical relationships is modulated by the cognitive, memory, and attentional states of the learners (Krogh et al. 2013), it is also possible that the developmental changes in perceptual/cognitive processes might explain the reported age differences in performance in SL tasks among ASD individuals. Indeed, most of the studies with older children (range: 8–17 years of age; Haebig et al. 2017; Mayo and Eigsti 2012; Roser et al. 2015) did not find group differences between ASD and controls, while studies with younger children (range: 3–months-8 years; Jeste et al. 2015; Jones et al. 2018; Marin et al. 2020) often reported impaired performance in ASD compared to TD participants. Accordingly, some studies have documented atypical profiles in young children with ASD that do not always persist across development (e.g. Davidson and Weismer 2017; Hudry et al. 2014). Finally, heterogeneity in early language development trajectories may also account for the observed differences in SL performance across age (Norbury et al. 2017; Pickles et al., 2014). Research on neurodevelopmental disorders has amply shown that performance in the normal range does not necessarily entail typical developmental trajectories and neural processes (see review by Karmiloff-Smith 2009). Therefore, the question of whether impairments in SL mechanisms may play a role in the etiology of the autistic behavior phenotype remains open, and the present findings from preverbal infants are an important contribution to answering such questions.

Our data revealed that lower SL abilities are associated with higher autistic traits (total AQ score). Given that the total AQ score measures social and communicative competencies (Baron-Cohen et al. 2001; Ruta et al. 2012), the present findings support the idea that visual SL is related to difficulties in the social-communicative domain underlying the BAP (Ruffman et al. 2012; Sinha et al. 2014). Indeed, social interaction is governed not only by verbal cues but also by unspoken rules. It has been claimed that impairments in the ability to detect and learn these non-verbal implicit rules from the environment may have a dramatic impact on the ability to understand and predict others' behavior and develop adequate social skills (Frith 1970a, 1970b; Klinger et al. 2007; Obeid et al. 2016). In line with this idea, recent evidence showed that inter-individual variations in visual SL skills are related to core ASD social symptoms: ASD children who perform better in visual SL tasks show higher adaptive social functioning (Jeste et al. 2015; Jones et al. 2018). Thus, early difficulties in detecting statistical patterns hidden in the visual input may have disruptive cascading effects on complex nonverbal communication skills rooted in the ability to pick up regularities in non-verbal cues from social interaction. Future studies should further explore this issue exploring whether difficulties in detecting visual statistical patterns may have a negative effect on complex nonverbal communication skills that might be linked to the ability to pick up non-verbal cues from social interaction.

Our data further highlights the potential benefit of considering the broader autism phenotype (BAP) as a model to understand ASD (Landry and Chouinard 2016). First of all, infants at risk to develop BAP by having a parent with sub-threshold high autistic traits share common alterations in low-level cognitive processes with infant siblings of ASD children (e.g. visual attention, Ronconi et al. 2014; alpha-band brain oscillations, Riva et al 2019; visual SL, our study), suggesting that the BAP approach is crucial since infancy to evaluate the presence of endophenotypes related to ASD symptoms. Moreover, autistic traits can be evaluated in the general population giving the possibility to understand the functioning of the autistic phenotype with many more participants than in most studies with ASD children or infants at risk for ASD. However, outcome measures derived from concurrent evaluation or follow-up studies are needed to determine how early disfunction in the low cognitive process, such as SL abilities, contributes to inter-individual variability of ASD symptoms.

Overall, in line with a neuroconstructivist view of human development (Karmiloff-Smith 1998), the present study highlights the relevance of investigating implicit learning processes in infants at risk to develop intellectual and developmental disabilities (e.g. Autism Spectrum Disorders, Developmental Language Disorders, and Williams Syndrome; Saffran 2018). Neuroconstructivism posits that complex cognitive abilities, such as language and social cognition, emerge as the result of complex and continuous interactions between different factors, e.g., genes, cognitive processes, and environment. Tiny variations in the initial state of the system, such as the atypical SL reported in the current study, might cause a cascading effect on the developing system, resulting in distinct clinical outcomes later in development.
